# Antifungal effects of Lactobacillus acidophilus and Lactobacillus plantarum against different oral Candida species isolated from HIV/ AIDS patients: an in vitro study

**DOI:** 10.1080/20002297.2020.1769386

**Published:** 2020-05-25

**Authors:** Samira Salari, Pooya Ghasemi Nejad Almani

**Affiliations:** a Medical Mycology and Bacteriology Research Center, Kerman University of Medical Sciences, Kerman, Iran; b Students Research Committee, Kerman University of Medical Sciences, Kerman, Iran; c Department of Medical Parasitology and Mycology, Kerman University of Medical Sciences, Kerman, Iran; d Leishmaniasis Research Center, Kerman University of Medical Sciences, Kerman, Iran

**Keywords:** Antifungal effects, probiotic, *L. acidophilus*, *L. plantarum*, *Candida* species, HIV/AIDS patients

## Abstract

Oropharyngeal Candidiasis (OPC) is an opportunistic fungal infection occurring in immunocompromised  patients such as HIV/AIDS. The purpose of this study was to evaluate the antifungal properties of *Lactobacillus acidophilus* and *Lactobacillus plantarum* on different *Candida* species isolated from oral cavity of HIV/AIDS patients compared to Fluconazole (FLC). In this study, the antifungal effects of both cells and cell-free supernatants (CFSs) of *L. acidophilus* and *L. plantarum* were investigated against different oral *Candida* species by co-aggregation, agar overlay interference and broth microdilution assays, respectively. Our results showed that the highest co-aggregation ratio of the two tested Lactic acid bacteria (LAB) was observed for *C. krusei*. Both *L. acidophilus* and *L. plantarum* at cell concentrations 10^10^ to 10^2^ cfu/ml were able to inhibit the growth of most of the oral *Candida* species, except for *C. albicans*, and to some *C. krusei*. In this study, MIC and MFC values for CFS of *L. acidophilus* ranged from 100 to 200 µl/ml and 100 to 200 µl/ml, respectively, and MIC and MFC values for CFS of *L. plantarum* were 50 to 200 µl/ml and 50 to 200 µl/ml, respectively. The ranges of MIC and MFC for FLC were 256–1024 µg/ml and 512–2048 µg/ml, respectively. *C. albicans* and *C. parapsilosis* displayed the highest and least susceptibility to CFSs of two LAB, respectively. Our findings showed that both cells and CFSs of *L. acidophilus* and *L. plantarum* had antifungal effects against oral *Candida* species.

## Introduction

Probiotics are live microorganisms that, when consumed in sufficient quantities can increase the microbial balance in the host’s gut and be beneficial to human health. The major probiotics include *Lactobacillus* spp, *Bacillus* spp, *Bifidobacterium* spp, *Escherichia coli*, and *Saccharomyces cerevisiae* [1]. Lactic acid bacteria (LAB) are known as major probiotics and are considered as a group of normal gram-positive microbiota living in the gastrointestinal tract mucosa. The colonization of these bacteria has a vital role in protection against pathogenic microorganisms [2,3].

*Lactobacillus acidophilus* and *Lactobacillus plantarum* are the most common species of *Lactobacillus* spp in the gut, and a number of these species are introduced as probiotics [4]. *Lactobacillus* species have the ability to produce several antimicrobial substances including hydrogen peroxide, acetic acid, lactic acid, bacteriocins such as small heat-stable lantibiotics (SHSL), non-lanthionine-containing membrane-active peptides (MAP), larger heat-labile proteins (LHLP), and complex bacteriocins containing one or several of chemical components. Because of the ability to produce various antimicrobial agents, these probiotics could be candidates for the control and treatment of different infections [5].

Oropharyngeal Candidiasis (OPC) is known as an opportunistic fungal infection in immunocompromised patients  [6]. *Candida albicans* is the most common cause of OPC. Moreover, other *Candida* species such as *C. tropicalis, C. glabrata, C. krusei, C. kefyr, C. parapsilosis*, and *C. dubliniensis* have been isolated from infected areas in the mouth [7,8]. The different clinical signs of OPC in HIV/ADIS patients include oral thrush (pseudomembranous candidiasis), linear gingival erythema, erythematous candidiasis, perleche or angular cheilitis, salivary gland swellings, sore formation in the oral cavity, and oral hairy leukoplakia [9].

At present, development of resistant fungal strains and treatment failures following high or long-term use of antifungal  drugs have increased in immunocompromised patients [10,11]. Therefore, finding an alternative bio-ecological method for better control and treatment of fungal infections has been suggested [12]. The aim of the present study was to investigate the ability of *L. acidophilus* and *L. plantarum* to inhibit the growth of different oral *Candida* species isolated from HIV/AIDS patients under *in* *vitro* conditions.

## Materials and methods

### Probiotic species and culture conditions

Two *lactobacillus* species, *L. acidophilus* and *L. plantarum* were used in this study. These species generously provided by Dr Hamid Frootanfar from the Department of Pharmaceutical Biotechnology, Faculty of Pharmacy, Kerman University of Medical Sciences, Kerman, Iran. The two LAB species were initially cultured on De Man-Rogosa-Sharpe (MRS) agar (Liofilchem Company, Italy) at 37°C for 24 h in anaerobic conditions. Detached colonies of each LAB species were transferred to 5 ml MRS broth (Liofilchem Company, Italy), and then incubated in a shaker incubator at 37°C for 48 h. At the end of incubation time, two LAB species were kept in glycerol stocks at −20°C until use. For recultivation, 1 ml of *L. acidophilus* and *L. plantarum* stock were added to 5 ml MRS broth medium. Fifty microliters L-cysteine was added and microtubes placed in a shaker incubator at 37°C for 20 h (Lab companion, South Korea) for 48 h at 37°C.

### Candida species and culture conditions

In this study, five different *Candida* species including *C. albicans, C. parapsilosis, C. glabrata, C. kefyr*, and *C. krusei* were used. These clinical *Candida* species isolated from oral cavity of HIV/AIDS patients and identified previously by the specific color the colony created on CHROMagar *Candida* media and PCR-RFLP with *Msp I* enzyme [9,13,14].

### Co-aggregation assay

The co-aggregation was determined spectrophotometrically by UV-VIS/VIS spectrophotometer AE-S60 (AELAB Company, Guangzhou, Guangdong, China) in mixtures *L. acidophilus* and *L. plantarum*, and suspensions of each *Candida* species after 1, 2 and 4 h incubation and presented as the aggregation ratio (%) according to Jørgensen et al. study [7]. Briefly, the detached colonies of each 24 h culture of *L. acidophilus and L. plantarum* were transferred to a sterile microtube containing 5 mL MRS broth and were incubated in a shaker incubator at 84 rpm for 24 h at 37°C in an anaerobic chamber. On the other hand, different five *Candida* species were collected from Sabouraud Dextrose Agar (Liofilchem Company, Italy) and incubated in Sabouraud Dextrose broth (Liofilchem Company, Italy) at 37°C for 24 h. After 24 h incubation, the microtubes containing two LAB and *Candida* species were centrifuged separately at 855 rpm (Eppendorf Company, Hamburg, Germany) for 10 min at 25°C. Obtained pellets washed carefully thrice in phosphate-buffered saline (PBS), and suspended in 10 mmol/L PBS (pH = 7.0). The absorbance rate was set to an optical density (OD) equivalent to a McFarland standard of 600 nm (approximately equal to 10^8^ cfu/ml for two LAB species and 10^6^ cfu/ml for each *Candida* species) using a UV-VIS/VIS spectrophotometer AE-S60. 1 ml of each the LAB and 1 ml of each *Candida* species were completely mixed and incubated in a shaker incubator at 100 rpm at 37°C for 1, 2, and 4 h without any stimulation. Prior to each OD measurement, the microtubes containing each LAB and *Candida* species mixture were completely vortexed for at least 10 s. After 4 h incubation at 37°C, the OD measurement was carried out using a spectrophotometer at OD_600_ _nm_. The experiments were performed in triplicate. Then, the co-aggregation percentage was calculated using the following formula [7,15]:

$$\%co-aggregation=\frac{oDo-oD_{h}}{oD_{o}}\times 100$$

where OD_0_ shows the absorption amount of the complex suspension of each LAB with each *Candida* species at the beginning of the experiment (0 h) and OD_h_ shows the absorption amount of the complex solutions at various times (1, 2, and 4 h).

### Agar overlay interference assay

The growth inhibition of five oral *Candida* species by *L. acidophilus* and *L. plantarum* was done base on Keller et al. study [16]. Briefly, one distinct colony of 24 h cultured two LAB was transferred to a sterile microtube containing 5 ml MRS broth and was incubated anaerobically at 37°C for one day. The next day, the LAB species were harvested by centrifugation for 10 minutes at 855 g. The supernatants of two LAB species culture were removed. Then, the pellets were washed thrice in PBS and transferred again to the MRS broth. Cell suspensions corresponding to approximately 10^10^, 10^8^, 10^6^, 10^4^, and 10^2^ cfu/ml of *L. acidophilus* and *L. plantarum* were made. 1 ml of different cell concentrations of two LAB (10^10^ cfu/ml to 10^2^ cfu/ml) was added to 24 ml sterilized molten MRS agar (approximately 45°C) in petri dishes. When the medium became solid, the plates were anaerobically incubated at 37°C for 24 h. After incubation, 24 ml of sterilized molten sabouraud dextrose agar (approximately 45°C) were added to the top of the MRS agar layer containing cultured two LAB. The plates were kept at room temperature for 3 hours to solidify. 40 µl of cell suspension equivalent to 10^6^ cfu/ml from each *Candida* species was distributed on top of sabouraud dextrose agar with a sterilized steer’s replicator and was left to dry. The plates were placed at room temperature (approximately 24–25.5 °C) for one hour and incubated for one day at 37°C in an anaerobic chamber. As controls, each *Candida* species was distributed on top of sabouraud dextrose agar on the plate containing MRS agar layer without two LAB. All experiments were performed in triplicate. The obtained results were evaluated based on Simark-Mattsson *et al*. study [17]. A score of 0 = Full containment (no visible colonies), Score 1 = partial inhibition (at least one colony is visible but certainly smaller than the control plate), and Score 2 = without containment (similar growth with the control plate).

### Susceptibility of different Candida species to FLC, CFSs of L. acidophilus and L. plantarum

#### Preparation of Cell-free supernatants (CFSs) of L. acidophilus and L. plantarum

*L. acidophilus and L. plantarum* were grown into MRS broth and held at 37°C for 24 h. On the next day, the MRS broth containing each LAB species centrifuged for 10 min at 12,000 rpm at 4°C. Cells of *L. acidophilus* and *L. plantarum* were removed and the CFSs of two LAB species were harvested. Each CFS of LAB was filtered via a 0.22 µm sterilized syringe-driven filter (Jet Biofil, Guangdong, China) [18–20]. The CFSs of two LAB were kept at −20°C until use.

#### Evaluation of antifungal activities of Cell-free supernatants (CFSs) of L. acidophilus and L. plantarum and FLC using broth microdilution (BMD) method

The minimum inhibitory concentration (MIC) values of the CFSs of *L. plantarum* and *L. acidophilus* and FLC  against five different *Candida* species were determined by broth microdilution (BMD) based on the guidelines of the CLSI M27-S4 document [21]. The BMD assay was done using RPMI 1640 (Sigma Aldrich, USA) buffered with MOPS (Sigma Chemical Co.) in a 96 microtiter plate (Greiner, Germany). FLC powder (Sigma Aldrich, USA) was dissolved in Dimethyl sulfoxide (DMSO) (Merck, Germany). Different concentrations in the range of 200–0.781 µl/ml for CFSs of *L. plantarum* and *L. acidophilus* and 2048–0.0625 μg/ml for FLC were made in RPMI 1640 medium. Then, a suspension containing 1.5 × 10^3^ cells/ml of each *Candida* species was added to all the wells. Then, the plates were incubated in a shaking incubator at 100 rpm at 37°C for 24 h. MIC values for FLC, CFSs of *L. acidophilus* and *L. plantarum* were calculated using a microplate reader (BioTek Co, USA) at 570 nm. The lowest concentration of FLC, CFSs of *L. acidophilus* and *L. plantarum*, which reduces 90% in turbidity in comparison with the growth of control well considered as MIC value. All the tests were carried out in triplicate. Finally, average results for MICs were presented as µl/ml for two LAB species and μg/ml for FLC, respectively. The minimum fungicidal concentration (MFC) was considered as the lowest concentration for FLC, CFSs of *L. acidophilus* and *L. plantarum*, which were able to kill ≥99.9% of the five *Candida* species. Briefly, 10 μl of the wells with invisible growth were transferred to SDA plates. Then, the plates were incubated for 24 h at 35°C. The lowest amount of FLC, CFSs of *L. acidophilus* and *L. plantarum*, that created three colonies or less in the SDA medium was determined as MFC values [10,22].

#### Statistical Analysis

The results of susceptibility of different *Candida* species to FLC, CFSs of *L. acidophilus* and *L. plantarum* were presented as µl/ml for two LAB and μg/ml for FLC, respectively. These data analyzed by Graph Pad Prism version 8 (Graph Pad Software In, San Diego, USA) and ANOVA multiple comparison test. Data analysis on co-aggregation assay was done using student’s t-test. Results of agar overlay interference assay were analyzed by the chi-square test and expressed as the median inhibition score. The significance rate for all experiments was considered p < 0.05.

## Results

### Co-aggregation percentage between L. acidophilus and L. plantarum with different oral Candida species

**Figure 1.**
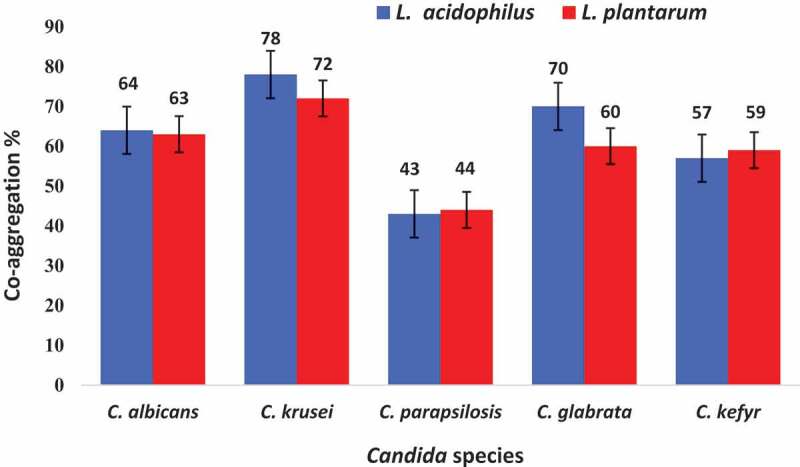
Average of co-aggregation degrees (%) between *L. acidophilus* and *L. plantarum* with five oral *Candida* species after 4 hours incubation. Error bars indicate standard deviations.

The co-aggregation results after 4 h are demonstrated in percentages (%) in Figure 1. Both *L. acidophilus* and *L. plantarum* species had co-aggregation ability with different oral *Candida* species with varying degrees. Co-aggregation percentage enhanced significantly with increase in time (p < 0.05). *L. acidophilus* displayed the highest co-aggregation ratio for *C. krusei* (78%) followed by *C. glabrata* (70%) after 4 h incubation. The co-aggregation ratio ranking of *L. acidophilus* with the tested five *Candida* species was *C. krusei > C. glabrata> C. albicans > C. kefyr > C. parapsilosis*. The highest co-aggregation ratio of *L. plantarum* was observed with *C. krusei* (72%), followed by *C. albicans* (63%) and *C. glabrata* (60%). The co-aggregation degree ranking of *L. plantarum* with different oral *Candida* species was: *C. krusei > C. albicans > C. glabrata > C. kefyr > C. parapsilosis*.

The co-aggregation score of *L. acidophilus* and *L. plantarum* for *C. albicans, C. parapsilosis*, and *C. kefyr* were approximately equal. No statistically significant differences were observed between the *L. acidophilus* and *L. plantarum* species for these three *Candida* species. A statistically significant differences (p < 0.05) were observed in co-aggregation ratios of *L. acidophilus* and *L. plantarum* with *C. krusei* and *C. glabrata*.

### Growth inhibition of five oral Candida species by L. acidophilus and L. plantarum

Table 1 shows growth inhibition of five oral *Candida* spp isolated from HIV/ADIS patients with OPC at different cell concentrations of *L. acidophilus* and *L. plantarum*. At high cell concentrations (10^10^ cfu/ml and 10^8^ cfu/ml), both *L. acidophilus* and *L. plantarum* inhibited the growth of all tested *Candida* spp. At cell concentrations 10^6^ cfu/ml and 10^4^ cfu/ml, the two LAB species showed slight inhibition on the five *Candida* spp. Also, at lower cell concentrations (10^2^ cfu/ml), a slight inhibition in growth of *C. glabrata, C. kefyr* and *C. parapsilosis* by *L. acidophilus* and for *C. glabrata, C. kefyr, C. parapsilosis*, and *C. krusei* by *L. plantarum* were observed, respectively. *L. acidophilus* displayed no inhibition for *C. albicans* and *C. krusei* at cell concentrations 10^2^ cfu/ml, and no growth inhibition was viewed only for *C. albicans* by *L. plantarum* at this concentration. Overall, at concentrations 10^10^ cfu/ml to 10^2^ cfu/ml, no statistically significant differences were observed between inhibitory effects of two both *L. acidophilus* and *L. plantarum* on *Candida* species except *C. krusei*. At in concentration 10^2^ cfu/ml, *L. plantarum* displayed superiority at inhibiting *C. krusei* compared *L. acidophilus* (p < 0.05).

**Table 1.** Growth inhibition of five oral *Candida* spp by *L. acidophilus* and *L. plantarum* at different cell concentrations.

***Candida*****species**	***L. acidophilusL. plantarum***									
	**cfu/ml**									
	**10^10^**	**10^8^**	**10^6^**	**10^4^**	**10^2^**	**10^10^**	**10^8^**	**10^6^**	**10^4^**	**10^2^**
***C.albicans***	0	0	1	1	2	0	0	1	1	2
***C.krusei***	0	0	1	1	2	0	0	1	1	1
***C.parapsilosis***	0	0	1	1	1	0	0	1	1	1
***C.glabrata***	0	0	1	1	1	0	0	1	1	1
***C.kefyr***	0	0	1	1	1	0	0	1	1	1

A score of 0 = Full containment (no visible colonies), Score 1 = partial inhibition (at least one colony is visible, but certainly smaller than the control plate), and Score 2 = without containment (similar growth with the control plate).

### Susceptibility of different oral Candida species to FLC, Cell-free supernatants of L. acidophilus and L. plantarum

**Figure 2.**
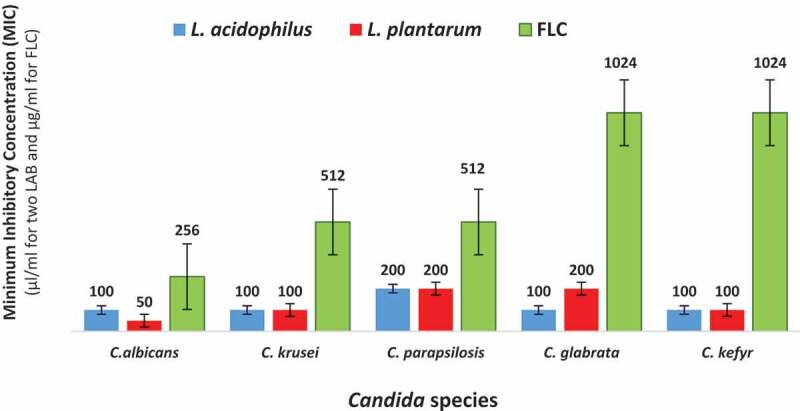
Minimum inhibitory concentrations (MIC) values of CFSs of *L. plantarum* and *L. acidophilus,* compared to FLC against five *Candida* species. Error bars indicate standard deviations.

**Figure 3.**
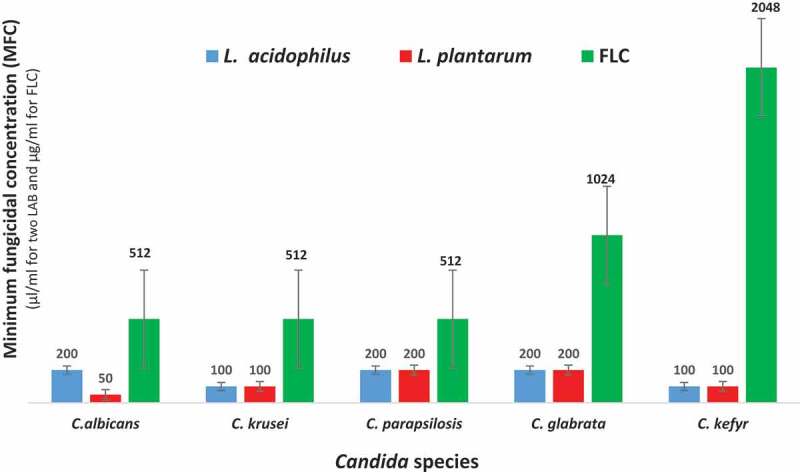
Minimum fungicidal concentrations (MFC) values of CFSs of *L. plantarum* and *L. acidophilus*, compared to FLC against five *Candida* species. Error bars indicate standard deviations.

Figures 2 and 3 show MIC and MFC values for CFSs of *L. acidophilus, L. plantarum*, compared to FLC on five different *Candida* species. In this study, MIC and MFC values for CFS of *L. acidophilus* ranged from 100 to 200 µl/ml and 100 to 200 µl/ml, respectively, and MIC and MFC values for CFS of *L. plantarum* were 50 to 200 µl/ml and 50 to 200 µl/ml, respectively. The range of MIC and MFC values for FLC were 256–1024 µg/ml and 512–2048 µg/ml, respectively.

### Comparison of inhibitory effects FLC, Cell-free supernatants of L. acidophilus and L. plantarum on different oral Candida species

The CFS of *L. acidophilus* displayed equal inhibitory effects on *C. albicans, C. krusei, C. kefyr*, and *C. glabrata*. The susceptibility ranking of *Candida* spp to the CFS of *L. acidophilus* was: *C. albicans, C. krusei, C. kefyr* and *C. glabrata > C. parapsilosis*. The CFS of *L. plantarum* inhibited the growth of *C. albicans* significantly, followed by *C. krusei* and *C. kefyr* (p < 0.05). The susceptibility ranking of *Candida* spp to the CFS of *L. plantarum* was: *C. albicans> C. krusei* and *C. kefyr> C. glabrata* and *C. parapsilosis*. Generally, *C. albicans* and *C. parapsilosis* displayed the highest and least susceptibility to CFSs of two LAB, respectively.

The susceptibility ranking of *Candida* species to FLC was: *C. albicans* > *C. krusei* and *C. parapsilosis* > *C. kefyr* and *C. glabrata*. Therefore, *C. albicans* showed the highest sensitivity to FLC among the tested *Candida* species. The lowest inhibitory effect of FLC was found on *C. kefyr* and *C. glabrata*. For all tested *Candida* spp, the antifungal effects of *L. acidophilus* and *L. plantarum* were higher than FLC among five oral *Candida* species (p < 0.05).

### Comparison of fungicidal effects of FLC, Cell-free supernatants of L. acidophilus and L. plantarum on different oral Candida species

Comparison of fungicidal effects of supernatants of *L. acidophilus* and *L. plantarum* and FLC was shown in Figure 3. The fungicidal effects ranking of *Candida* spp to the CFS of *L. acidophilus* was: *C. krusei* and *C. kefyr > C. albicans* and *C. parapsilosis> C. glabrata*. The CFS of *L. acidophilus* had highest fungicidal effects on *C. krusei* and *C. kefyr* (p < 0.05). The lowest lethal effect of CFS of *L. acidophilus* was found on *C. glabrata*. The fungicidal effects ranking of *Candida* spp to the CFS of *L. plantarum* was: *C. albicans > C. krusei* and *C. kefyr > C. glabrata* and *C. parapsilosis*. Generally, *C. albicans* and *C. glabrata* and *C. parapsilosis* displayed the highest and least lethal effects to CFS of *L. plantarum*, respectively. The fungicidal effects ranking of *Candida* species to FLC was: *C. albicans, C. krusei* and *C. parapsilosis* > *C. glabrata* > *C. kefyr*. The fungicidal effects of FLC were equal on *C. albicans, C. krusei* and *C. parapsilosis. C. kefyr* showed the lowest lethal effects to FLC among the tested *Candida* species. The fungicidal effects of *L. acidophilus* and *L. plantarum* were higher than FLC for five different *Candida* spp (p < 0.05).

### Comparison of susceptibilities of different Candida species to supernatants of L. acidophilus and L. plantarum and FLC

The antifungal effects of supernatants of *L. acidophilus* and *L. plantarum* on *Candida* species were compared to FLC. For *C. albicans*, the inhibitory effect of *L. plantarum* CFS is higher than the CFS of *L. acidophilus* (p < 0.0036). In addition, the inhibitory properties of the CFSs of two LAB species were greater than FLC. The difference between the growth inhibition of *C. albicans* by the CFSs of two LAB and FLC was significant (p < 0.001). For *C. glabrata*, the most intense inhibition was observed at low concentrations of *L. acidophilus* CFS compared to CFS of *L. plantarum* and FLC (p < 0.0001). In addition, significant difference was detected between the antifungal effects of the CFSs of the two LAB species (p < 0.003). CFSs of *L. acidophilus* and *L. plantarum* exhibited equal antifungal activities against *C. krusei, C. parapsilosis* and *C. kefyr* (p > 0.999). However, for these three species, the CFSs of the two LAB species had a significantly greater inhibitory effect on *Candida* growth than FLC (p < 0.0001).

## Discussion

Due to increase in incidence of candidiasis in immunocompromised patients, development of resistance in *Candida* spp to current antifungal agents, the frequent relapses of this disease and failures in the treatment of candidiasis, the use of some useful compounds such as probiotics for control and treatment of this fungal infection can be suggested as an interesting therapeutic strategy [1]. In general, the antimicrobial activity of LAB species is well known [23]. Various investigators have demonstrated anticandidal effects of different LAB species including *L. acidophilus* [24], *L. plantarum* [25], *L. paracasei* [26], *L. rhamnosus* [15], *L. reuteri* [7], *L. casei* [27], and other clinical isolates of *Lactobacillus*. In this study, the antifungal effects of both cells and CFSs of *L. acidophilus* and *L. plantarum* were investigated against different oral *Candida* species by co-aggregation, agar overlay interference and broth microdilution methods, respectively.

Various studies have shown a different rate in the co-aggregate scores of different LAB species with the tested *Candida* species. In present study, *C. krusei* and *C. parapsilosis* showed the highest and lowest co-aggregation degree with *L. acidophilus* and *L. plantarum*, respectively. Here, the most of co-aggregation percent’s of *L. acidophilus* and *L. plantarum* with *C. krusei* followed by *C. glabrata* were observed significantly greater than co-aggregation score than those reported in the study performed by Jørgensen *et al*. [7], which showed that both *L. reuteri* strains had the highest co-aggregation ratio with *C. tropicalis* and *C. krusei*. In addition, in their study, *L. reuteri*  ATCC PTA 5289 exhibited stronger co-aggregation ratio for all the tested *Candida* spp compared to *L. reuteri* DSM 17938. While, here, higher co-aggregation level of *L. acidophilus* with *C. krusei, C. glabrata* and *C. tropicalis* higher than L. *plantarum*. Contrary to our results, *L. plantarum* 319 showed the maximum aggregation with *C. glabrata* and *C. albicans* [28].

In contrast, an another study demonstrated that *L. crispatus* had the highest co-aggregation degrees with *C. tropicalis, C. glabrata, C. albicans*, and *C. krusei* [29], and Chew et al. [15] reported that *L. reuteri* RC-14 displayed a particularly higher co-aggregation level versus all the tested *C. glabrata* species in comparison with *L. rhamnosus* GR-1. It seems that the co-aggregation levels is specific and unique for each species of *Lactobacillus*.

Various studies have demonstrated that the lactobacilli have antifungal effects on different *Candida* species. Agar overlay interference is a simple and dependable way for the evaluation of antifungal properties of different probiotics against *Candida* species. The advantage of this method is feasibility for different concentrations of probiotics within a plate [7]. In this study, both *L. acidophilus* and *L. plantarum* at cell concentrations 10^10^ to 10^2^ Cfu/ml were able to inhibit the growth of most of the oral *Candida* species, except for *C. albicans*, and to some *C. krusei*. In the study concluded by Jørgensen *et al*., both *L. reuteri* strains exhibited good inhibitory effects on the growth of most of the tested *Candida* spp, except for *C. tropicalis* and *C. krusei* [7]. Similar to our finding, Jiang *et al*. [30] and Zhao *et al*. [31] reported that the lactobacilli failed to inhibit *C. krusei*. Contrary to the present study, *C. albicans* was the most susceptible yeast to lactobacilli [30]. Hasslöf *et al*. reported that at cell concentrations 10^9^ and 10^7^ cfu/ml of LAB species, except *L. reuteri* PTA 5289 and *L. acidophilus* La5, inhibition of *Candida* species growth was observed by other probiotics. In their study, at cell concentration 10^5^ cfu/ml, *L. reuteri* PTA 5289, *L. rhamnosus* GG ATCC 53103, *L. rhamnosus* LB21, and *L. paracasei* F19 exhibited week inhibition properties, and *L. acidophilus* La5 had no inhibitory effect. However, *L. plantarum* 931, *L. plantarum* 299v, and *L. reuteri* ATCC 55730  demonstrated strong inhibition. Similar to our study, at low cell concentration (10^3^ cfu/ml) of LAB strains cells, except for *L. plantarum* strain, no growth inhibition was observed [3].

In another part of this study, we examined the antifungal effects of CFSs of *L. acidophilus* and *L. plantarum* at different concentrations on five oral *Candida* species. In this study, *C. albicans* was the most susceptible to CFSs of two LAB. Here, MIC and MFC values for CFS of *L. acidophilus* ranged from 100 to 200 μL/ml. These values greater than those reported by Aminnezhad *et al.* [32], who reported that the growth of *P. aeruginosa* was inhibited by CFSs of *L. casei* and *L. rhamnosus* at concentration of 62.5 μl/ml. Coman *et al.* showed that the most of the pathogenic yeasts and bacteria were inhibited by *L. rhamnosus* and *L. paracase* with various degrees [26].

Lower antibacterial effects for CFSs of *L. acidophilus* LA5 and *L. casei* 431 compared to our study was reported by Koohestani *et al.* [19]. Contrary to our results, a strong antifungal activity of *L. pentosus* strain LAP1 was observed versus *C. tropicalis*, followed by *C. albicans* and *C. krusei* [33]. CFSs of *L. gasseri* and *L. rhamnosus* inhibited the mixed biofilms of non-*albicans Candida* species and damaged the cells [34]. Cell-free supernatant of *L. acidophilus* was inhibited biofilm development and filamentation of *C. albicans* [24]. The differences in results of different studies may be related to differences in the examined lactobacilli strains, the experiments for evaluating antifungal effects, examined *Candida* species, the initial counts of LAB species, the duration of incubation, and the origin of the *Candida* spp isolation.

The mechanism of action of *Lactobacillus* strains as an effective probiotic is related to the presentation of a 29 kD collagen-binding protein on the surface and the production of biosurfactants such as surlactin that allow them to prevent the binding and decampment of harmful microorganisms into different tissues of the host’s body, reduction in luminal pH, and the production of H_2_O_2_, which is toxic for harmful microorganisms. Stimulation of innate and adaptive immune responses includes the synthesis of inflammatory cytokines, producing various antimicrobial substances including hydrogen peroxide, acetic acid, lactic acid, bacteriocins such as small heat-stable lantibiotics (SHSL), non-lanthionine-containing membrane-active peptides (MAP), larger heat-labile proteins (LHLP), and complex bacteriocins containing one or several of chemical components are number of mechanisms suggested for the action of probiotics [1,35,36]. It is noteworthy that these mechanisms vary in different species of lactobacillus.

A potentially interesting and novel aspect of this study is the comparison of antifungal effects of both cells and CFSs of *L. acidophilus* and *L. plantarum* on different species using clinical isolates. These clinical species involved *C. albicans, C. parapsilosis, C. glabrata, C. kefyr*, and *C. krusei* that isolated from oral cavity of HIV/AIDS patients. During HIV infection period, the incidence of candidiasis is related to reduce the immunity level of these patients due to decreased CD_4_^+^ cells, which is dependent on the use of antiviral therapy [37]. *C. albicans, non-albicans Candida* species and *Cryptococcus neoformans* are the most common yeasts isolated from HIV/AIDS patients [38]. One limitation of the present study is the lack of investigation of the possible antifungal effects of *L. acidophilus* and *L. plantarum* on some species such as *C. dubliniensis, C. tropicalis* and *C. guilliermondi.*

## Conclusion

Both cells and CFSs of *L. acidophilus* and *L. plantarum* showed antifungal effects against the five oral *Candida* species. Our finding revealed that both *L. acidophilus* and *L. plantarum* at cell concentrations 10^10^ to 10^2^ cfu/ml was able to inhibit the growth of most of the oral *Candida* species, except for *C. albicans*, and to some C. krusei. Here, *C.* *albicans* and *C. parapsilosis* displayed the highest and least susceptibility to CFSs of two LAB, respectively. Considering the obtained results and importance of candidiasis in immunocompromised hosts, treatment failures due to formation of resistant species, and the side effects of chemical drugs, further investigations for evaluating of the antifungal properties of *L. acidophilus* and *L. plantarum* and other *lactobacillus* species, identifying the exact mechanisms of their action, and performing antifungal studies in infected experimental animals are suggested.

## References

[1] Silva MP, Rossoni RD, Junqueira JC, et al. Probiotics for Prevention and Treatment of Candidiasis and Other Infectious Diseases: Lactobacillus spp. and Other Potential Bacterial Species. In: Rao V, Rao L, eds. Probiotics and Prebiotics in Human Nutrition and Health. IntechOpen, London, UK, 2016; 242–262.

[2] Kheradmand E, Rafii F, Yazdi MH, et al. The antimicrobial effects of selenium nanoparticle-enriched probiotics and their fermented broth against Candida albicans. DARU J Pharma Sci. 2014;22:1–9. 10.1186/2008-2231-22-48 PMC4060857 24906455

[3] Hasslöf P, Hedberg M, Twetman S, et al. Growth inhibition of oral mutans streptococci and candida by commercial probiotic lactobacilli-an in vitro study. BMC Oral Health. 2010;10:1–6. 20598145 10.1186/1472-6831-10-18 PMC2908555

[4] Gudadappanavar AM, Hombal PR, Timashetti SS, et al. Influence of Lactobacillus acidophilus and Lactobacillus plantarum on wound healing in male Wistar rats-an experimental study. Int J App Basic Med Res. 2017;7:233–238. 10.4103/ijabmr.IJABMR_329_16 PMC5752807 29308360

[5] Spinler JK, Taweechotipatr M, Rognerud CL, et al. Human-derived probiotic Lactobacillus reuteri demonstrate antimicrobial activities targeting diverse enteric bacterial pathogens. Anaerobe. 2008;14:166–171. 18396068 10.1016/j.anaerobe.2008.02.001 PMC2494590

[6] Salari S, Khosravi AR, Mousavi SAA, et al. Mechanisms of resistance to fluconazole in Candida albicans clinical isolates from Iranian HIV-infected patients with oropharyngeal candidiasis. Journal de mycologie medicale. 2016;26:35–41. 26627124 10.1016/j.mycmed.2015.10.007

[7] Jørgensen MR, Kragelund C, Jensen PØ, et al. Probiotic Lactobacillus reuteri has antifungal effects on oral Candida species in vitro. J Oral Microbiol. 2017;9:1–8. 10.1080/20002297.2016.1274582 PMC5328390 28326154

[8] Barati M, Mirkalantari S, Ansari S, et al. Determination of antimicotic susceptibility pattern of Candida species isolated from patients with symptomatic candiduria. J Res Med Sci. 2019;24:1–3. 31143236 10.4103/jrms.JRMS_880_18 PMC6521611

[9] Pour AH, Salari S, Ghasemi Nejad Almani P. Oropharyngeal candidiasis in HIV/AIDS patients and non-HIV subjects in the Southeast of Iran. Curr Med Mycol. 2018;4:1–6. 10.18502/cmm.4.4.379 PMC6386505 30815610

[10] Salari S, Bakhshi T, Sharififar F, et al. Evaluation of antifungal activity of standardized extract of Salvia rhytidea Benth. (Lamiaceae) against various Candida isolates. Journal de mycologie medicale. 2016;26:323–330. 27499461 10.1016/j.mycmed.2016.06.003

[11] Fallah Zahabi Z, Sharififar F, Ghasemi Nejad Almani P, et al. Antifungal activity of different fractions of Salvia rhytidea Benth as a valuable medicinal plant against various species of Candida in Kerman Province, southeast Iran. Gene Rep. 2020;19:1–7.

[12] Seddighi NS, Salari S, Izadi AR. Evaluation of antifungal effect of iron-oxide nanoparticles against different Candida species. IET Nanobiotechnol. 2017;11:883–888.

[13] Bakhshi T, Salari S, Naseri A, et al. Molecular identification of Candida species in patients with candidiasis in Birjand, Iran, using polymerase Chain reaction-restriction fragment length polymorphism (PCR-RFLP) assay. J Isfahan Med Sch. 2016;33:1986–1993.

[14] Ayatollahi Mousavi SA, Salari S, Rezaei S, et al. Identification of Candida species isolated from oral colonization in Iranian HIV-positive patients, by PCR-RFLP method. Jundishapur J Microbiol. 2012;5:336–340.

[15] Chew S, Cheah Y, Seow H, et al. Probiotic L actobacillus rhamnosus GR‐1 and L actobacillus reuteri RC‐14 exhibit strong antifungal effects against vulvovaginal candidiasis‐causing C andida glabrata isolates. J Appl Microbiol. 2015;118:1180–1190. 25688886 10.1111/jam.12772 PMC4406132

[16] Keller MK, Hasslöf P, Stecksén-Blicks C, et al. Co-aggregation and growth inhibition of probiotic lactobacilli and clinical isolates of mutans streptococci: an in vitro study. Acta Odontol Scand. 2011;69:263–268. 21306197 10.3109/00016357.2011.554863

[17] Simark‐Mattsson C, Emilson CG, Håkansson EG, et al. Lactobacillus‐mediated interference of mutans streptococci in caries‐free vs. caries‐active subjects. Eur J Oral Sci. 2007;115:308–314. 17697171 10.1111/j.1600-0722.2007.00458.x

[18] Bulgasem BY, Lani MN, Hassan Z, et al. Antifungal activity of lactic acid bacteria strains isolated from natural honey against pathogenic Candida species. Mycobiology. 2016;44:302–309. 28154488 10.5941/MYCO.2016.44.4.302 PMC5287163

[19] Koohestani M, Moradi M, Tajik H, et al. Effects of cell-free supernatant of Lactobacillus acidophilus LA5 and Lactobacillus casei 431 against planktonic form and biofilm of Staphylococcus aureus. Vet Res Forum. 2018;9:301–306. 30713607 10.30466/vrf.2018.33086 PMC6346487

[20] Mariam SH, Zegeye N, Tariku T, et al. Potential of cell-free supernatants from cultures of selected lactic acid bacteria and yeast obtained from local fermented foods as inhibitors of Listeria monocytogenes, Salmonella spp. and Staphylococcus aureus. BMC Res Notes. 2014;7:1–9. 25190588 10.1186/1756-0500-7-606 PMC4167124

[21] Clinical and Laboratory Standards Institute. Reference method for broth dilution antifungal susceptibility testing of yeasts: 4th informational supplement CLSI M27-S4. Wayne, PA 19087 USA; 2012.

[22] Uraipan S, Hongpattarakere T. Antagonistic characteristics against food-borne pathogenic bacteria of lactic acid bacteria and bifidobacteria isolated from feces of healthy Thai infants. Jundishapur J Microbiol. 2015;8:1–9. 10.5812/jjm.8(5)2015.18264 PMC4539568 26301060

[23] Strus M, Kucharska A, Kukla G, et al. The in vitro activity of vaginal Lactobacillus with probiotic properties against Candida. Infect Dis Obstet Gynecol. 2005;13:69–75. 16011996 10.1080/10647440400028136 PMC1784560

[24] Vilela SF, Barbosa JO, Rossoni RD, et al. Lactobacillus acidophilus ATCC 4356 inhibits biofilm formation by C. albicans and attenuates the experimental candidiasis in Galleria mellonella. Virulence. 2015;6:29–39. 25654408 10.4161/21505594.2014.981486 PMC4603435

[25] Biyari S, Fozouni L. The inhibitory effect of probiotic bacteria against drug-resistant Candida species isolated from the oral cavity of the elderly. Shiraz E-Med J. 2018;19:2–7.

[26] Coman M, Verdenelli M, Cecchini C, et al. In vitro evaluation of antimicrobial activity of L actobacillus rhamnosus IMC 501®, L actobacillus paracasei IMC 502® and SYNBIO® against pathogens. J Appl Microbiol. 2014;117:518–527. 24836638 10.1111/jam.12544

[27] Mendonça FHBP, Santos S, Faria I, et al. Effects of probiotic bacteria on Candida presence and IgA anti-Candida in the oral cavity of elderly. Braz Dent J. 2012;23:534–538. 23306230 10.1590/s0103-64402012000500011

[28] Verdenelli M, Coman M, Cecchini C, et al. Evaluation of antipathogenic activity and adherence properties of human L actobacillus strains for vaginal formulations. J Appl Microbiol. 2014;116:1297–1307. 24552173 10.1111/jam.12459

[29] Gil NF, Martinez RC, Gomes BC, et al. Vaginal lactobacilli as potential probiotics against Candida spp. Braz J Microbiol. 2010;41:6–14. 24031455 10.1590/S1517-83822010000100002 PMC3768620

[30] Jiang Q, Stamatova I, Kari K, et al. Inhibitory activity in vitro of probiotic lactobacilli against oral Candida under different fermentation conditions. Benef Microbes. 2014;6:361–368. 10.3920/BM2014.0054 25380800

[31] Zhao C, Lv X, Fu J, et al. In vitro inhibitory activity of probiotic products against oral Candida species. J Appl Microbiol. 2016;121:254–262. 26999745 10.1111/jam.13138

[32] Aminnezhad S, Kermanshahi RK, Ranjbar R. Evaluation of synergistic interactions between cell-free supernatant of Lactobacillus strains and amikacin and genetamicin against Pseudomonas aeruginosa. Jundishapur J Microbiol. 2015;8:1–9. 10.5812/jjm.8(4)2015.16592 PMC4449849 26034539

[33] Aarti C, Khusro A, Varghese R, et al. In vitro investigation on probiotic, anti-Candida, and antibiofilm properties of Lactobacillus pentosus strain LAP1. Arch Oral Biol. 2018;89:99–106. 29499562 10.1016/j.archoralbio.2018.02.014

[34] Tan Y, Leonhard M, Moser D, et al. Inhibitory effect of probiotic lactobacilli supernatants on single and mixed non-albicans Candida species biofilm. Arch Oral Biol. 2018;85:40–45. 29031236 10.1016/j.archoralbio.2017.10.002

[35] Bermudez-Brito M, Plaza-Díaz J, Muñoz-Quezada S, et al. Probiotic mechanisms of action. Ann Nutr Metab. 2012;61:160–174. 23037511 10.1159/000342079

[36] Raheja G, Singh V, Ma K, et al. Lactobacillus acidophilus stimulates the expression of SLC26A3 via a transcriptional mechanism. Am J Physiol Gastrointest Liver Physiol. 2009;298:G395–G401. 20044511 10.1152/ajpgi.00465.2009 PMC2838518

[37] Kaur R, Dhakad MS, Goyal R, et al. Spectrum of opportunistic fungal infections in HIV/AIDS patients in tertiary care hospital in India. Can J Infect Dis Med Microbiol. 2016;2016:1–7. 10.1155/2016/2373424 PMC4931070 27413381

[38] Anwar KP, Malik A, Subhan KH. Profile of candidiasis in HIV infected patients. Iran J Microbiol. 2012;4:204–209. 23205253 PMC3507311

